# Clinical Potential of Targeting Fibroblast Growth Factor‐23 and αKlotho in the Treatment of Uremic Cardiomyopathy

**DOI:** 10.1161/JAHA.120.016041

**Published:** 2020-03-26

**Authors:** Jonathan P. Law, Anna M. Price, Luke Pickup, Ashwin Radhakrishnan, Chris Weston, Alan M. Jones, Helen M. McGettrick, Winnie Chua, Richard P. Steeds, Larissa Fabritz, Paulus Kirchhof, Davor Pavlovic, Jonathan N. Townend, Charles J. Ferro

**Affiliations:** ^1^ Birmingham Cardio‐Renal Group University Hospitals Birmingham University of Birmingham United Kingdom; ^2^ Institute of Cardiovascular Sciences University of Birmingham United Kingdom; ^3^ Institute of Immunology and Immunotherapy University of Birmingham United Kingdom; ^4^ School of Pharmacy University of Birmingham United Kingdom; ^5^ Institute of Inflammation and Ageing University of Birmingham United Kingdom; ^6^ NIHR Birmingham Biomedical Research Centre University Hospitals Birmingham NHS Foundation Trust and University of Birmingham United Kingdom; ^7^ Department of Cardiology University Hospitals Birmingham NHS Foundation Trust Birmingham United Kingdom; ^8^ Department of Nephrology University Hospitals Birmingham NHS Foundation Trust Birmingham United Kingdom

**Keywords:** αKlotho, cardiorenal syndrome, FGF23, fibroblast growth factor, growth factor, kidney, treatment, Cardiorenal Syndrome, Growth Factors/Cytokines, Treatment

## Abstract

Chronic kidney disease is highly prevalent, affecting 10% to 15% of the adult population worldwide and is associated with increased cardiovascular morbidity and mortality. As chronic kidney disease worsens, a unique cardiovascular phenotype develops characterized by heart muscle disease, increased arterial stiffness, atherosclerosis, and hypertension. Cardiovascular risk is multifaceted, but most cardiovascular deaths in patients with advanced chronic kidney disease are caused by heart failure and sudden cardiac death. While the exact drivers of these deaths are unknown, they are believed to be caused by uremic cardiomyopathy: a specific pattern of myocardial hypertrophy, fibrosis, with both diastolic and systolic dysfunction. Although the pathogenesis of uremic cardiomyopathy is likely to be multifactorial, accumulating evidence suggests increased production of fibroblast growth factor‐23 and αKlotho deficiency as potential major drivers of cardiac remodeling in patients with uremic cardiomyopathy. In this article we review the increasing understanding of the physiology and clinical aspects of uremic cardiomyopathy and the rapidly increasing knowledge of the biology of both fibroblast growth factor‐23 and αKlotho. Finally, we discuss how dissection of these pathological processes is aiding the development of therapeutic options, including small molecules and antibodies, directly aimed at improving the cardiovascular outcomes of patients with chronic kidney disease and end‐stage renal disease.

**Nonstandard Abbreviations and Acronyms**
AF: atrial fibrillation
ESRD: end‐stage renal disease
FGF23: fibroblast growth factor 23
LV: left ventricular
LVH: left ventricular hypertrophy
SCD: sudden cardiac death

## Introduction

Chronic kidney disease (CKD) and end‐stage renal disease (ESRD) requiring dialysis are complex, chronic conditions with a combined prevalence of 10% to 15% of the adult population worldwide.^1^, ^2^, ^3^ Cardiovascular events and mortality increase exponentially with reduced estimated glomerular filtration rate (eGFR) independent of age, sex, and other risk factors.^4^, ^5^, ^6^ In the early stages of CKD, the risks of occlusive atheromatous disease are increased and account for the majority of cardiovascular events observed.^7^ Arterial atheroma remains an important modifiable pathophysiological process in CKD, as evidenced by trials in early CKD showing benefit from lipid‐lowering therapies in modifying the risk of atherosclerotic events.^8^, ^9^, ^10^ However, the same treatments appear much less effective in patients with advanced stages of CKD, including ESRD.^10^, ^11^, ^12^ As CKD worsens, there is a shift from atherosclerotic complications to morbidity due to heart failure and sudden cardiac death (SCD).^7^, ^13^, ^14^ Atrial fibrillation (AF) is also common, detected in up to 41% of patients requiring hemodialysis.^14^ The pathophysiological basis of these events is a unique cardiovascular phenotype consisting primarily of the development of uremic cardiomyopathy with associated increased arterial stiffness and widespread atheroma.

The purpose of this article is to review the current state of the art on 2 newly postulated drivers of uremic cardiomyopathy, elevated circulating fibroblast growth factor‐23 (FGF23) and reduced αKlotho, and discuss how recent insights into the pathophysiological processes has led to development of potential therapeutic options aimed at reducing the cardiovascular risk of patients with CKD/ESRD.

## Uremic Cardiomyopathy

The term uremic cardiomyopathy arose in the 1980s with reports of common abnormalities in cardiac function and structure in patients with CKD/ESRD, including increased left ventricular (LV) mass and left ventricular hypertrophy (LVH); diastolic and systolic dysfunction; as well as profound myocardial fibrosis on histology.^15^, ^16^, ^17^, ^18^, ^19^, ^20^ Uremic cardiomyopathy has been linked to conditions causing both heart failure and CKD, especially hypertension. Chronic stimulation of cardiac cells by renin, angiotensin, parathyroid hormone (PTH), cardiotonic steroids, and other uremic toxins, has also been proposed.^20^ The severity of uremic cardiomyopathy as measured by LV mass is a powerful predictor of cardiovascular mortality probably as a result of the factors discussed below.

### Increased LV Mass and Hypertrophy

Increased LV mass and LVH are common manifestations of uremic cardiomyopathy. Forty percent of patients with an eGFR <30 mL/min per 1.73 m^2^ have LVH on echocardiography,^21^ increasing to ≈80% in ESRD.^22^, ^23^ In patients with CKD/ESRD, LVH is strongly associated with death; diastolic and systolic heart failure; and cardiac arrhythmias.^22^, ^23^ However, LV mass is a continuous variable, with a graded relationship with adverse cardiovascular outcomes.^24^, ^25^, ^26^, ^27^, ^28^ It is also important to emphasize that cardiac structural changes occur early in the course of CKD, with a linear association between worsening renal function and a higher prevalence of LVH.^29^, ^30^ While elevated blood pressure is an important determinant of LV mass,^31^ evidence from both animal and human studies supports the presence of mechanisms that are independent of pressure overload and hypertension in driving cardiac hypertrophy in CKD/ESRD.^32^, ^33^, ^34^, ^35^, ^36^

### Diastolic and Systolic Dysfunction

Diastolic dysfunction is highly prevalent in patients with CKD, with over two thirds affected in CKD stages 2 to 4^30^ and up to 85% in ESRD.^37^ Diastolic dysfunction is strongly associated with increased LV mass and LVH,^7^ as well as myocardial fibrosis,^20^, ^38^ and correlates with increased mortality.^37^, ^38^ Furthermore, the presence of diastolic dysfunction is considered to be a major cause for the frequent presentation of hemodialysis patients with pulmonary edema or intradialytic hypotension, despite only minor changes in fluid status.^7^, ^38^

Overt LV systolic dysfunction, as manifested by reduced ejection fraction, is uncommon in predialysis CKD with a reported prevalence of 8% and no association with eGFR.^17^, ^30^ However, several studies using echocardiography have shown changes in LV deformation in early stages of CKD, indicating the presence of subnormal LV systolic function.^39^, ^40^, ^41^ In ESRD, LV systolic dysfunction is very common, with a reported prevalence 10 to 30 times greater than in the general population.^42^, ^43^

### Myocardial Fibrosis

It has been suggested that increased interstitial myocardial fibrosis may be the unifying pathophysiological process underlying uremic cardiomyopathy.^44^ In the 1990s, a postmortem study found that myocardial fibrosis was present in 91% of CKD/ESRD patients without significant flow‐limiting coronary lesions. The severity of fibrosis was related to the length of time on dialysis, but independent of hypertension, blood pressure, diabetes mellitus, or anemia.^15^ Over a decade later, Aoki et al.^16^ performed endocardial biopsies in 40 ESRD patients with reduced LV ejection fraction without coronary artery disease. The predominant pathologic findings were extensive interstitial fibrosis and cardiomyocyte hypertrophy and disarray.

Studying myocardial fibrosis in CKD/ESRD has been challenging given that myocardial biopsies are not without risk, especially in multimorbid patients and therefore are not always clinically and ethically justified.^45^, ^46^ Late gadolinium enhancement cardiac magnetic resonance imaging, a validated noninvasive method, allows in vivo quantification of myocardial fibrosis in conditions such as myocardial infarction,^47^ dilated^48^ and hypertrophic cardiomyopathies.^49^ This technique has also been used to characterize myocardial tissue in patients with ESRD demonstrating midwall patterns of late gadolinium enhancement consistent with replacement myocardial fibrosis not associated with large vessel coronary artery disease.^17^ Noncontrast myocardial native T1 relaxation time, or T1 mapping, has emerged as a novel viable technique to quantify diffuse interstitial myocardial fibrosis in CKD/ESRD^50^ correlating with histological interstitial fibrosis in a number of disease states, including cardiomyopathy and valvular disease.^51^ Indeed, native T1 times are increased in early CKD,^19^ increasing with worsening CKD stages^52^ and correlates with increased LV mass.^18^, ^53^ Native T1 mapping offers an exciting opportunity to investigate novel mechanisms of cardiac fibrosis (eg, FGF23‐mediated changes), in patients with CKD and in animal models.

## Fibroblast Growth Factor‐23 and αKlotho

**Figure 1:**
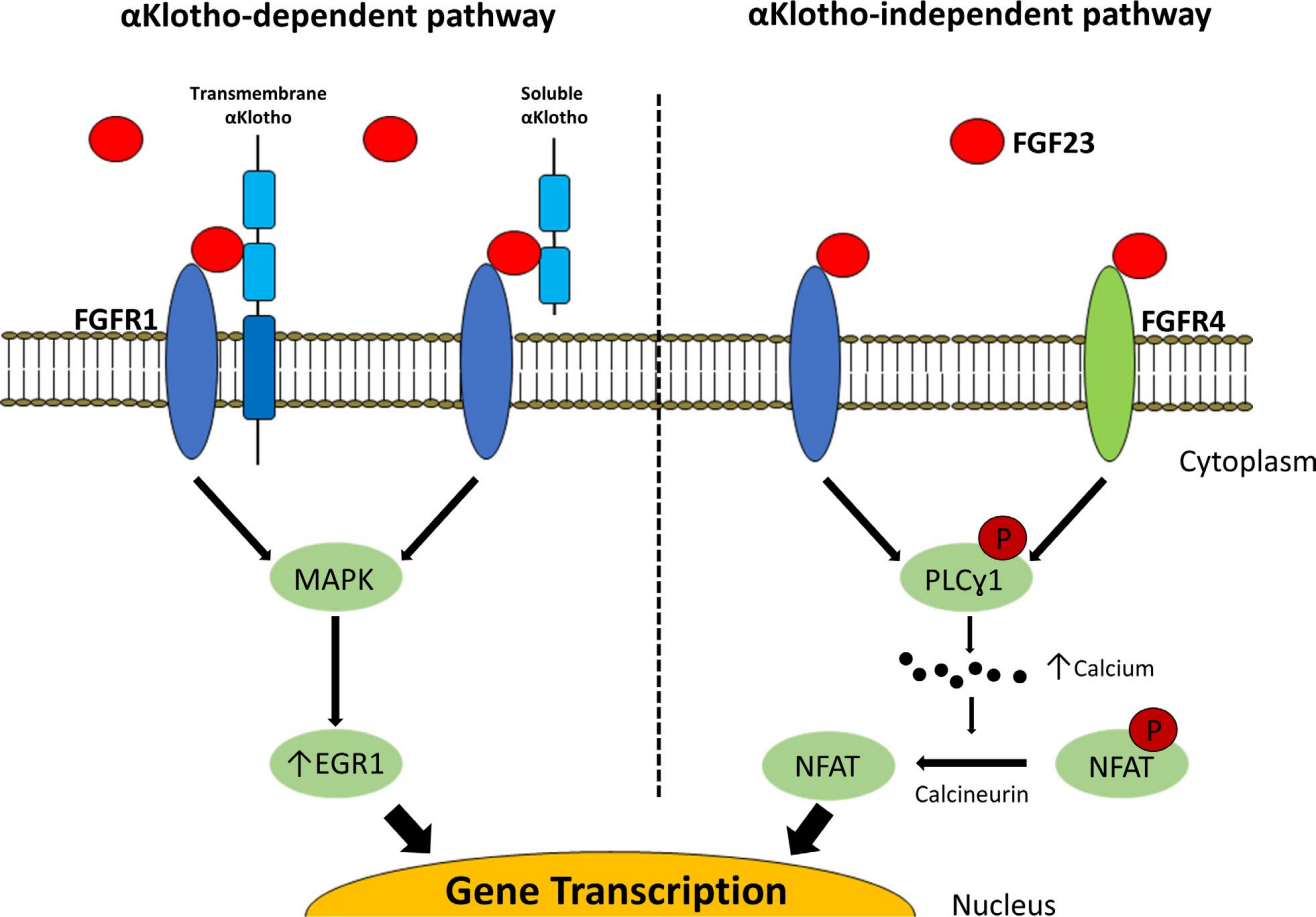
Potential mechanisms of fibroblast growth factor‐23 (FGF23) signal transduction and signaling pathways. Fibroblast growth factor‐23 (FGF23) acts on cells that constitutively express fibroblast growth factor receptor‐1 (FGFR1) and its coreceptor αKlotho. In cells that do not constitutively express αKlotho, such as cardiac myocytes and fibroblasts, circulating αKlotho is thought to perform a role similar to membrane‐bound αKlotho. Circulating FGF23 can also bind to other FGFR1 and other FGFR independently of αKlotho. In cardiac myocytes, this is thought to be FGFR4 predominantly. The binding of FGF23 to an FGFR1‐αKlotho complex activates MAPK (mitogen‐activated protein kinase), upregulating early growth response protein‐1 (EGR1), thereby modulating physiological gene expression. In the absence of αKlotho, FGF23 binding to either FGFR1 or FGFR4 activates phospholipase Cγ1 (PLCγ1), increasing intracellular calcium. This in turn activates calcineurin to dephosphorylate (P, in red) nuclear factor of activated T‐cells (NFAT), which induces pathophysiological gene transcription.

The hormone FGF23, first discovered in 2000, is a circulating growth factor secreted by osteocytes whose main physiological role is to increase urinary phosphate excretion.^54^, ^55^ The 4 mammalian FGF receptors (FGFR1‐4) are membrane‐bound receptor tyrosine kinases.^56^, ^57^ FGFR1 is suggested to be the primary FGF23 receptor in target organs—the kidneys and parathyroid glands.^58^, ^59^, ^60^, ^61^ Crystallography studies clearly demonstrate that the presence of αKlotho is required for the efficient binding of FGF23 to FGFR1.^62^ αKlotho is a cell‐surface protein, mainly expressed in the kidneys and parathyroid glands.^63^, ^64^, ^65^ In addition to the membrane‐associated full‐length protein, the ectodomain of αKlotho can exist in a soluble form.^66^, ^67^, ^68^, ^69^ In the presence of membrane‐bound αKlotho or soluble αKlotho, FGF23 can activate Fibroblast Growth Factor Receptor Substrate‐2 (FRS2α)/Ras/Mitogen‐Activated Protein Kinase signaling (Figure 1).^62^, ^70^, ^71^ Soluble αKlotho, therefore, may act as a circulating FGF23 coreceptor in cells that do not express αKlotho. Such a mechanism has been reported in osteoblasts.^72^ However, its role in the heart is yet to be fully characterized as neither cardiomyocytes nor cardiac fibroblasts express αKlotho.^55^ It is possible that FGF23 might act on these cells with circulating αKlotho as a cofactor. Treatment of cardiac myofibroblasts with full‐length αKlotho resulted in upregulated proliferation and ERK phosphorylation, which was suppressed by FGFR1 antagonism.^73^ This suggests the presence of FGFR1 in cardiac myofibroblasts for which soluble Klotho acts as a circulating co‐receptor, although the authors did not comment on endogenous FGFR1/FGF23 expression.

FGF23 can also exert cellular effects via αKlotho‐independent mechanisms.^74^ FGF23 has been shown to stimulate phospholipase Cγ (PLCγ)/calcineurin/nuclear factor of activated T‐cells (NFAT) via FGFR4 in cells that lack αKlotho (Figure 1).^74^, ^75^, ^76^, ^77^ Such increases in PLCγ/calcineurin/NFAT signaling appear to be important in pathological, as opposed to physiological, cardiac hypertrophy.^78^, ^79^ Clearly further mechanistic studies are warranted to delineate mechanisms that can be targeted therapeutically in patients with elevated FGF23 levels.

## FGF23, αKlotho, and Kidney Disease

**Figure 2:**
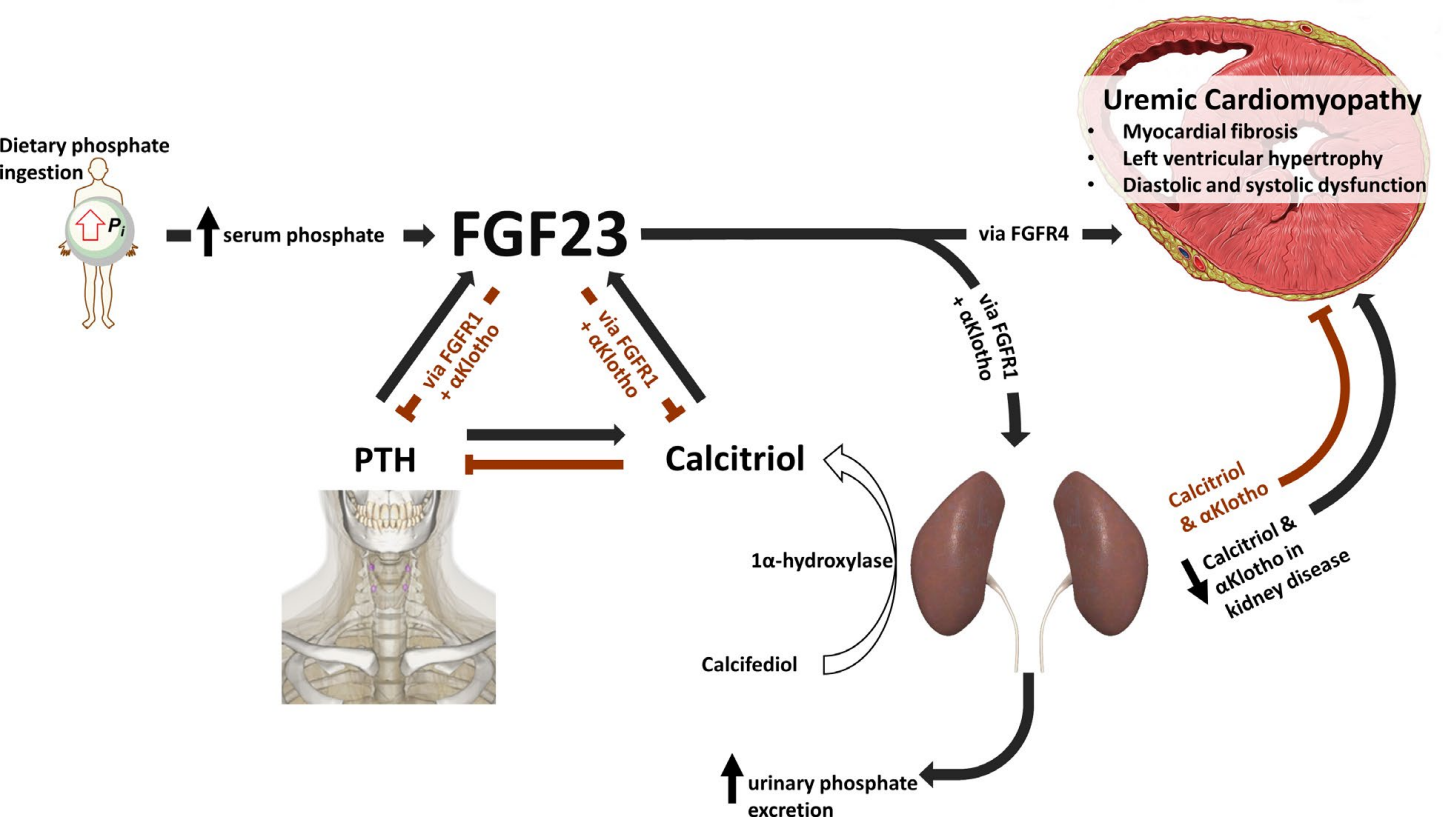
Dynamic interplay of fibroblast growth factor‐23–αKlotho axis. Dietary phosphate (Pi) ingestion and absorption leads to osteocytes secreting fibroblast growth factor‐23 (FGF23). It is not clear how osteocytes detect circulating phosphate, but the sensing of calciprotein particles (nanoparticles consisting of calcium, phosphate, and fetuin A) has been proposed as a potential mechanism. Increased FGF23 leads to increased phosphate excretion by downregulating sodium‐dependent phosphate cotransporter IIa/c, via mitogen‐activated protein kinase signaling, in the renal proximal tubules expressing FGF‐receptor 1 (FGFR1) in an αKlotho‐dependent manner. In addition, FGF23 lowers renal vitamin D hydroxylation and decreases parathyroid hormone (PTH) secretion, both actions also being αKlotho‐dependent, reducing calcium entry into the circulation. In the context of reducing renal function, FGF23 production increases to compensate for reduced renal phosphate excretion, decreased activated vitamin D levels, and rising PTH secretion. High levels of circulating FGF23 have been implicated in the development of uremic cardiomyopathy through αKlotho‐independent mechanisms via either the FGFR1 or, more likely, FGFR4. Circulating αKlotho may mitigate some of the pathophysiological actions of FGF23 on the myocardium. However, the kidneys are the main source of circulating αKlotho and levels decrease with reducing renal function.

One of the first clinically detectable signs of CKD is an elevation in serum FGF23, probably in response to increased extracellular phosphate, although the details of the stimulus and its detection are still unclear, with levels rising steeply as kidney function worsens^54^, ^80^ (Figure 2). Indeed, elevations are observed as early as eGFR 75 mL/min per 1.73 m^2^, long before increased concentrations in PTH or phosphate are observed.^80^ Circulating FGF23 levels are 2‐ to 5‐fold above the normal range in early/intermediate CKD, but can reach levels of 1000‐fold above normal in ESRD.^80^, ^81^, ^82^ Increased FGF23 levels are also found in heart failure^83^, ^84^, ^85^, ^86^, ^87^, ^88^ and AF,^89^, ^90^, ^91^, ^92^, ^93^ and are associated with all‐cause and cardiovascular mortality in patients with and without CKD.^87^, ^88^, ^94^, ^95^, ^96^, ^97^, ^98^, ^99^ Ongoing research therefore explores FGF23 as both a potential biomarker^100^ and a causative factor for cardiac mechanoelectrical dysfunction. However, effective quantification of circulating FGF23 is not currently standardized. Several FGF23 assay kits utilize differing detection techniques, epitope binding regions, analytical ranges and measurement units, making direct comparisons challenging.^101^

The kidney is the principal source of circulating soluble αKlotho.^69^, ^102^ Its levels are downregulated in the presence of albuminuria,^103^ inflammation,^104^ and with the progression of CKD. αKlotho levels start to decline in CKD stage 2 and precede the elevation of FGF23, PTH, and serum phosphate.^105^ Low levels of circulating αKlotho are associated with increased cardiovascular events and mortality in patients with CKD/ESRD.^106^, ^107^, ^108^, ^109^ It is, therefore, conceivable that some of the adverse physiological effects that have been attributed to increased FGF23 may be either caused by, or compounded by, lower αKlotho (Figure 2). These mechanistic complexities require further investigation and need to be considered when developing FGF23/αKlotho‐directed therapies.

## FGF23, αKlotho, and Left Ventricular Mass/Hypertrophy

The heart has been shown to respond to FGF23, increasing LV mass independently of blood pressure, promoting cardiac fibrosis and reducing LV systolic function in animal models.^74^, ^75^, ^77^, ^110^, ^111^ Elevations of cardiac FGFR4 and enhanced PLCγ/calcineurin/NFAT signaling have been observed in both animal models of CKD and in patients with CKD/ESRD.^75^, ^112^, ^113^, ^114^ Several studies have shown that repetitive administration of FGF23 in wild‐type mice, either intravenous or intraperitoneal, induced cardiac hypertrophy within 5 days.^74^, ^110^, ^111^ The signaling actions of FGF23 on the heart are still not fully characterized. However, several independent experimental approaches demonstrate the involvement of the αKlotho‐independent FGFR4‐PLCγ/calcineurin/NFAT signaling pathway in cardiomyocytes.^74^, ^75^, ^77^ On the other hand, independent studies demonstrate that αKlotho may be cardioprotective and that subnormal levels may be required for FGF23 to induce LVH.^115^, ^116^, ^117^, ^118^ Although many of the studies indicate that elevation of FGF23 and reduction in αKlotho are involved in the development of LVH, recent studies by Leifheit‐Nestler and Slavic have not recapitulated these findings.^119^, ^120^ Chronic FGF23 overexpression (via myocardial gene transfer), or genetic ablation of FGF23 or αKlotho on the background of transverse aortic constriction did not affect cardiac function or morphology.^119^, ^120^

## Fibroblast Growth Factor‐23, αKlotho, and Myocardial Fibrosis

Every third to fourth cell in the heart is a fibroblast. Fibroblasts produce the extracellular matrix in the heart and act as regulators of the cardiac interstitium.^121^, ^122^ In the injured myocardium, inflammation and mechanical stress promote activation of fibroblasts to myofibroblasts, leading to maladaptive deposition of extensively cross‐linked extracellular matrix, which drives increased stiffness and impaired mechanoelectrical coupling of cardiomyocytes. This loss of cardiomyocyte coupling not only leads to attenuated cardiac function, but also provides a substrate for arrhythmias.^123^, ^124^, ^125^, ^126^ Although recent studies have shown that FGF23 can activate cardiac fibroblasts, neither the underlying mechanism^127^, ^128^ nor its role in the development of cardiac fibrosis are fully defined.^127^, ^129^ As cardiac fibroblasts do not express αKlotho,^74^, ^102^, ^113^, ^114^ the alternative pathway of αKlotho‐independent FGF23 signaling through FGFR4‐PLCγ/calcineurin/NFAT is likely to play a role (Figure 1). Future studies are required to examine which FGF receptors are expressed in cardiac fibroblasts, whether FGF23 contributes to cardiac fibrosis, and whether these mechanisms are dependent on αKlotho.

## FGF23, αKlotho, and Cardiac Arrhythmias

Patients with CKD/ESRD are at increased risk of a wide spectrum of cardiac arrhythmias, including supraventricular tachycardias, particularly AF, and potentially lethal ventricular arrhythmias.^14^, ^130^, ^131^ All 3 components of uremic cardiomyopathy (increased LV mass/LVH; diastolic and systolic dysfunction; and especially myocardial fibrosis) are associated with arrhythmogenesis.^131^, ^132^ While emerging evidence from implantable loop recorder studies is beginning to implicate bradyarrhythmias as the major cause of SCD in ESRD, rather than the previously assumed tachyarrhythmias, the precise causes of SCD in ESRD are the subject of investigation.^14^ αKlotho has been found in sinoatrial node pacemaker cells in mice^133^ and αKlotho‐deficient animals exhibit sinoatrial node dysfunction, and higher rates of bradyarrhythmias and SCD.^133^

FGF23 distrupts intracellular calcium cycling within the cardiomyocyte, which is an important risk factor for arrhythmogenesis.^134^, ^135^, ^136^ Administration of FGF23 to rat ventricular cardiomyocytes caused calmodulin‐dependent protein kinase II‐dependent aberrant intracellular calcium, resulting in in vitro and in vivo arrhythmogenicity.^137^ Administration of recombinant αKlotho or a pan‐FGFR blocker prevented contractile dysfunction and reduced pro‐arrhythmogenic activity.^137^

Large observational studies in patients with CKD or AF and in the general population have found an association between elevated FGF23 and increased risk of developing AF.^89^, ^138^ High FGF23 and low αKlotho levels are associated with periods of AF in patients with paroxysmal or persistent AF.^91^ Increased expression of FGF23, FGFR4 mRNA, and FGFR4 protein in the right atrial appendages of patients with AF has been reported and positively correlate with atrial collagen fraction. Collectively these data/studies suggest that FGF23/FGFR4 may play a role in promoting AF through atrial fibrosis.^139^

## Reversing or Preventing Uremic Cardiomyopathy by Targeting the FGF23 and αKlotho Axis

Several therapies exist that directly or indirectly target FGF23, αKlotho, and abnormalities in bone metabolism. These are reviewed below and summarized in Table.

**Table 1:** Potential Therapies for Reversing or Preventing Uremic Cardiomyopathy by Targeting the Fibroblast Growth Factor‐23 and αKlotho Axis

Treatment	Study	Species	CKD Status	Outcome
Targeting phosphate				
Dietary phosphate restriction	Burnett et al^141^	Human	No renal impairment	Reduction in serum FGF23
	Antoniucci et al^143^	Human	No renal impairment	
	Moe et al^144^	Human	CKD Stage 3B to 4	
	Di Iorio et al^145^	Human	CKD Stage 3A to 4	
	Sigrist et al^146^	Human	No CKD & Stage 3A to 4	
	Rodriguez‐Ortiz et al^147^	Rat	5/6 Nx	
Calcium‐sparing phosphate binders (eg, sevelamer)	Oliveira et al^148^	Human	CKD Stage 3A to 4	
	Block et al^149^	Human	CKD Stage 3B to 4	
	Chue et al^150^	Human	CKD Stage 3	
	Rodelo‐Haad et al^151^	Human	ESRD on HD	
	Sprague et al^152^	Human	ESRD on HD/PD	
Nicotinamide	Shahbazian et al^155^	Human	ESRD on HD	
Tenapanor	Block et al^157^	Human	ESRD on HD	
	Labonte et al^156^	Rat	5/6 Nx	
Combination therapy with lanthanum and nicotinamide	Ix et al^159^	Human	CKD Stage 3B to 4	No sustained reduction in serum FGF23
Targeting Vitamin D				
Calcitriol	Leifheit‐Nestler et al^114^	Rat	5/6 Nx	Reduction in LVH, cardiac FGF23 & FGFR4 expression, and NFAT/calcineurin activation
	Leifheit‐Nestler et al^114^	Rat (NRVM)	n/a	In vitro reduction in FGF23‐induced cardiomyocyte hypertrophy
Calcitriol & paricalcitol	Lau et al^163^	Mice	Partial renal ablation, phosphate loaded	Increase in serum αKlotho. No effect on renal/parathyroid αKlotho expression
Paricalcitol	Ritter et al^164^	Rat	5/6 Nx	Preservation of renal αKlotho, and increase in parathyroid αKlotho expression in uremia
Targeting parathyroid hormone				
Cinacalcet	Moe et al^87^	Human	ESRD on HD	Reduction in serum FGF23, cardiovascular death, SCD, and heart failure
	Charytan et al^173^	Human	CKD Stage 3A to 4	Reduction in FGF23 and PTH
	Chonchol et al^174^	Human	CKD Stage 3A to 4	Reduction in FGF23 and PTH; increase in hypocalcemia
Other indirect targets				
Intensified (daily) hemodialysis	Zaritsky et al^175^	Human	ESRD on HD	Reduction in FGF23 vs conventional hemodialysis
Renal transplantation	Barros et al^176^	Human	ESRD (⅘ on HD)	Reduction in FGF23 and phosphate
Treatment of iron deficiency (eg, ferric citrate)	Block et al^179^, ^180^	Human	CKD Stage 3A to 5	Reduction in serum FGF32
Inhibition of inflammation (eg, NFᴋB inhibitor)	Rodriguez‐Ortiz et al^147^	Rat	No renal impairment	Attenuation of LPS‐induced FGF23 elevation
ATII receptor blockade	Yoon et al^181^	Mice	CsA‐induced renal injury	Increase in renal αKlotho expression
Statins (eg, atorvastatin, pitavastatin)	Narumiya et al^182^	Mouse (IMCD3)	n/a	In vitro upregulation of αKlotho mRNA expression
PPARγ agonist (eg, pioglitazone)	Yang et al^183^	Rat	No renal impairment	Increase in renal αKlotho expression
Exercise	Matsubara et al^184^	Human	No renal impairment	Increase in serum/plasma αKlotho
	Tan et al^185^			
Directly targeting FGF23				
FGF23 neutralizing antibodies	Hasegawa et al^189^	Rat	Anti‐GBM nephritis	Decrease in PTH; increase in vitamin D, calcium and phosphate
	Shalhoub et al^188^	Rat	5/6 Nx	In addition to above, increase in mortality & aortic calcification
FGFR antagonists				
FGFR4 antibody	Grabner et al^75^	Rat	5/6 Nx	Attenuation of LVH
		Rat (NRVM)	n/a	In vitro inhibition of FGF23‐induced cardiac myocyte hypertrophy
Pan‐FGFR antibody	Faul et al^74^	Rat	5/6 Nx	Attenuation of LVH
		Rat (NRVM)	n/a	In vitro inhibition of FGF23‐induced cardiac myocyte hypertrophy
	Di Marco et al^191^	Rat	5/6 Nx	Reduction in LV mass and fibrosis; improvement in ejection fraction
	Yanochko et al^192^	Rat	No renal impairment	Cardiac toxicity, hyperphosphatemia and ectopic calcification
Sodium‐phosphate co‐transporter PiT2 knockout	Bon et al^187^	Mice	No renal impairment	PiT2 regulates FGF23 synthesis; potential target for therapeutics
Directly targeting αKlotho				
Intravenous αKlotho transgene	Xie et al^198^	Mice	5/6 Nx±heterozygous Klotho	Attenuation of cardiac hypertrophy and fibrosis
Recombinant αKlotho	Hu et al^196^	Mice	Uni‐nephrectomy + contralateral IR injury	Preservation of cardiac function, reduced hypertrophy and fibrosis; attenuation of renal fibrosis
	Yang et al^118^	Mice	5/6 Nx	Inhibition of LVH and reduction in myocardial reactive oxygen species production
	Yu et al^200^	Mice	No renal impairment	Attenuation of angiotensin II‐induced cardiac hypertrophy, fibrosis, and dysfunction
	Suassuna et al^199^	Rat	5/6 Nx	Reduction of uremic cardiac remodeling (hypertrophy and fibrosis)
	Yang et al^118^	Rat (NRVM)	n/a	In vitro inhibition of uremic toxin‐induced (indoxyl sulphate) myocyte hypertrophy
Small molecule αKlotho modulators	King et al^201^	Human (HEK293)	n/a	In vitro elevation of αKlotho protein expression

5/6 Nx indicates 5/6 nephrectomized; anti‐GBM, anti‐glomerular basement membrane; ATII, angiotensin II; CKD, chronic kidney disease; CM, cardiomyocyte; CsA, cyclosporine A; eGFR, estimated glomerular filtration rate; ESRD, end‐stage renal disease; FGF23, fibroblast growth factor‐23; FGFR, fibroblast growth factor receptor; HD, hemodialysis; HEK293, human embryonic kidney 293 cells; IR, ischemia‐reperfusion; LPS, lipopolysaccharide; LV, left ventricle; LVH, left ventricular hypertrophy; n∕a, not applicable; NFAT, nuclear factor of activated T‐cells; NRVM, neonatal rat ventricular myocytes; PD, peritoneal dialysis; PPARγ, peroxisome proliferator‐activated receptor γ; PTH, parathyroid hormone; and SCD, sudden cardiac death.

### Targeting Phosphate Levels in the Body

Studies in healthy subjects have shown that circulating FGF23 levels are associated with dietary phosphate intake levels,^140^, ^141^ and can be further increased by acute phosphate loading.^142^ This can be reduced, in the short‐term, by aggressive reduction of dietary phosphate absorption^141^, ^143^, ^144^, ^145^, ^146^ and restriction.^147^ Overall, in relatively short‐term studies, noncalcium‐based phosphate binders lower FGF23 in patients with CKD/ESRD, whereas calcium‐based binders do not.^148^, ^149^, ^150^, ^151^, ^152^ Calcium is thought to be a secondary stimulus for FGF23 synthesis.^153^, ^154^ However, lowering intestinal phosphate absorption with dietary change, phosphate binders, nicotinamide,^155^ tenapanor,^156^, ^157^, ^158^ or combination therapy^159^ produces only modest decreases in FGF23 that do not appear to be sustained in the long term.^150^, ^159^ Whether this is because of increased total intestinal phosphate absorption by active phosphate transport,^160^, ^161^, ^162^ high pill burden, or intolerability of the medications is unknown.

### Targeting Vitamin D

There is strong experimental data supporting vitamin D as a potential treatment for FGF23‐mediated uremic cardiomyopathy. Calcitriol, the synthetic analogue of vitamin D_3_, blocks FGF23‐induced activation of FGFR4 and cardiomyocyte growth.^114^ Increases in FGF23 expression, FGFR4‐induced calcineurin/NFAT signaling, and LVH in 5/6 nephrectomized rats are reduced by calcitriol.^114^ Vitamin D also increases αKlotho expression.^163^, ^164^ Observational studies demonstrate a survival advantage of vitamin D therapy in patients with CKD/ESRD despite raising calcium and phosphate levels.^165^, ^166^ However, in a randomized, placebo‐controlled study in patients with CKD stages 4 to 5, paricalcitol (activated vitamin D_2_ analogue) treatment did not reduce LV mass.^167^ Taken together, these data suggest combining vitamin D receptor activation with FGF23/FGFR4 signaling blockade could have beneficial synergistic actions on uremic cardiomyopathy.

### Targeting Parathyroid Hormone

In patients on dialysis, the clinically available allosteric modulators (calcimimetics) of the calcium‐sensing receptor, cinacalcet and etelcalcetide, are used to treat hyperparathyroidism and consistently lower circulating FGF23.^168^, ^169^, ^170^, ^171^, ^172^ In secondary analyses of the large and well‐designed EVOLVE (Evaluation of Cinacalcet HCl Therapy to Lower Cardiovascular Events) trial, a >30% reduction in FGF23 in patients randomized to cinacalcet was associated with a reduction in cardiovascular mortality, SCD, and admissions for heart failure. The findings were amplified in those with a >50% reduction in FGF23.^87^

In CKD patients not requiring dialysis, randomized‐controlled trials of cinacalcet have reported significant reductions in FGF23, but also poor suppression of PTH as well as high rates of hypocalcemia and hyperphosphatemia.^173^, ^174^ These actions are thought to negate many of the clinical benefits of calcimimetics and these agents are not licensed for use in patients with non‐end‐stage CKD. Nevertheless, cinacalcet remains a promising therapeutic option for the treatment of uremic cardiomyopathy in ESRD.

### Other Indirect Targets

Intensified dialysis treatment,^175^ renal transplantation,^176^ reduced inflammation,^147^, ^177^, ^178^ and treatment of iron deficiency^152^, ^178^, ^179^, ^180^ all reduce circulating FGF23 levels. Angiotensin‐receptor antagonists,^181^ statins,^182^ peroxisome proliferator‐activated receptor gamma agonists,^183^ and exercise^184^, ^185^ all increase αKlotho expression. The clear indications for these treatments remain and may well continue to give further insights into the pathophysiology of FGF23, but it is unlikely that these interventions will be used to directly target FGF23 and αKlotho.

## Directly Targeting FGF23

The mechanism(s) regulating FGF23 synthesis are poorly understood and no “phosphate‐sensor” has yet been found in mammals.^186^, ^187^ Animal data have recently suggested that sodium‐phosphate cotransporter PiT2 found in bone might regulate phosphate‐dependent FGF23 synthesis and that targeting PiT2 could potentially reduce FGF23 synthesis.^187^ The development of novel small molecules against PiT2 or the yet‐to‐be characterized PiT2‐FGF23 pathway would give a proof of principle approach in animals for blocking FGF23 synthesis.

Indiscriminate FGF23 neutralization with monoclonal antibodies has been shown to worsen hyperphosphatemia, and increase vascular calcification and mortality in rat models of CKD.^188^, ^189^ Use of anti‐FGF23 monoclonal antibodies such as burosumab, currently approved for the treatment of x‐linked hypophosphatemia, causes severe side effects in patients with CKD by decreasing phosphaturia.^190^ Analogous to the use of calcimimetics, total blockade of FGF23 may theoretically be of benefit in ESRD. From a clinical therapeutic and drug development perspective, the ideal target would be the FGFR responsible for the adverse cardiac effects of FGF23 and not FGFR1, which is critical for maintaining normal phosphate levels. Indiscriminate blockade of FGFRs, although shown to be effective at preventing the development of,^191^ and reversing LVH in rodents,^74^ results in cardiac toxicity, hyperphosphatemia, and ectopic calcium deposition.^192^ Targeting cardiac FGFR4, especially its αKlotho‐independent activation of downstream signaling pathways, represents an exciting possibility. Indeed, FGFR4‐blocking antibodies have been shown to inhibit FGF23‐induced hypertrophy of isolated rat cardiomyocytes in vitro, and attenuated LVH in a 5/6 nephrectomy rat model of CKD.^75^ Currently, very little is known about the specific FGFRs mediating the actions of FGF23 in nonmyocyte cardiac cells including fibroblasts, or whether blocking FGFR4 prevents or reverses cardiac fibrosis.^128^ Several anti‐FGF small molecule tyrosine kinase inhibitors and related compounds, monoclonal antibodies, and FGFR‐analogues currently in development are mainly for use in oncology.^193^ Development of these agents specifically for the treatment of uremic cardiomyopathy is, therefore, a real possibility.

## Directly Targeting αKlotho

In animal studies, administration of αKlotho protein has been shown to be effective in protecting against progression of CKD.^194^, ^195^, ^196^, ^197^ Intravenous administration of a transgene‐encoding soluble αKlotho reduces LVH in αKlotho‐deficient mice.^198^ Recombinant αKlotho also attenuates cardiac remodeling, fibrosis,^195^, ^199^ reactive oxygen species production, and LVH^117^ induced by CKD in mice. In another study, αKlotho improved cardiac function and reduced hypertrophy and fibrosis in a mouse model of hypertension, although decreasing FGF23 expression.^200^ However, it remains unclear whether αKlotho is cardioprotective in the absence of increased FGF23.

Elevated FGF23 and decreased circulating αKlotho are observed in both aging and in CKD,^116^, ^197^, ^198^ leading to speculation that both CKD and the age‐related decline in this and other physiological functions are caused in part by increased FGF23 and decreased αKlotho.^55^ If true, this would assume soluble αKlotho acts as an inhibitor of αKlotho‐independent actions of FGF23. Potential mechanisms include inhibiting FGF23/FGFR4 signaling by either binding first to FGFR4 or via an initial interaction with FGF23 (decoy receptor). An alternative mechanism involves FGF23 and FGFR4 forming a complex in the presence of soluble αKlotho but activating FRS2α/Ras/mitogen‐activated protein inase rather than PLCγ/calcineurin/NFAT signaling (Figure 1).^55^ Therefore, development of αKlotho‐mimetics, through the development of protein–protein inhibitors, provides another potential therapeutic option. Trials of such agents to prevent progression of CKD are expected to start in the next couple of years.

To date, small molecule αKlotho modulators have been identified from a high‐throughput screen of 150 000 compounds with those showing most promise being αKlotho transcription activators. Furthermore, extracellular signal‐regulated kinase phosphorylation in FGFR‐transfected cells increased, demonstrating an effect on FGF23 signaling.^201^ The recently discovered crystal structure of αKlotho:FGF23:FGFR1 in a 1:1:1 relationship has provided new insight into this dynamic interplay of factors and may reveal new therapeutic options.^62^ Although clearly at an early stage of exploration, the identification of new small molecules demonstrates the potential of drugs acting via αKlotho.

## Conclusions

While patients with early stages of CKD are at increased risk of atherosclerotic complications, later stages of kidney disease are associated with heart failure and sudden death caused by uremic cardiomyopathy. Significant progress has been made over the past 2 decades in our understanding of, and ability to study the pathological basis of uremic cardiomyopathy using native T1 mapping. There are clear clinical data illustrating an association of increased FGF23 and reduced αKlotho with uremic cardiomyopathy in patients with CKD, and in heart failure and AF in subjects without known CKD. However, whether FGF23 has a truly causal relationship in uremic cardiomyopathy remains controversial. Characterization of the receptors and molecular pathways by which FGF23 might mediate LVH, cardiac fibrosis, and arrhythmias will help to identify therapeutic targets. Further work is required to identify the interplay between FGF23, cardiomyocytes and fibroblasts, and the effects of these interactions on the subsequent cardiac remodeling to reveal the molecular and cellular targets of FGF23 and αKlotho. Improved understanding is likely to enable the development of novel therapeutic interventions capable of effectively reducing the excess cardiovascular risk associated with CKD/ESRD, and perhaps even the risk of AF and heart failure in patients without CKD.

## Sources of Funding

This work was supported by the British Heart Foundation Accelerator Award (AA/18/2/34218 to Kirchhof and The Institute of Cardiovascular Sciences, University of Birmingham); British Heart Foundation Clinical Research Training Fellowships (FS/19/16/34169 to Law, FS/16/73/32314 to Price, and FS/18/29/44554 to Pickup); and British Heart Foundation Grants (PG/17/55/33087 to Pavlovic, RG/17/15/33106, to Pavlovic, FS/19/12/34204 to Pavlovic).

## Disclosures

None.
