# Correction to: Clinical Potential of Targeting Fibroblast Growth Factor‐23 and αKlotho in the Treatment of Uremic Cardiomyopathy

**DOI:** 10.1161/JAHA.119.014566

**Published:** 2020-07-17

In the article by Law et al, “Clinical Potential of Targeting Fibroblast Growth Factor‐23 and αKlotho in the Treatment of Uremic Cardiomyopathy,” which published online March 26, 2020, and appeared in the April 9, 2020 issue of the journal (*J Am Heart Assoc*. 2020;9:e016041. 10.1161/JAHA.120.016041), a correction was needed.

The article license was listed as, “© 2020 The Authors. Published on behalf of the American Heart Association, Inc., by Wiley. This is an open access article under the terms of the Creative Commons Attribution‐NonCommercial‐NoDerivs License, which permits use and distribution in any medium, provided the original work is properly cited, the use is non‐ commercial and no modifications or adaptations are made.” To comply with a funding mandate, the license has now been corrected to “© 2020 The Authors. Published on behalf of the American Heart Association, Inc., by Wiley. This is an open access article under the terms of the Creative Commons Attribution License, which permits use, distribution and reproduction in any medium, provided the original work is properly cited.”

The authors regret the error.

The online version of the article has been updated and is available at https://www.ahajournals.org/doi/10.1161/JAHA.120.016041

